# When the market got the first dose: Stock volatility and vaccination campaign in COVID-19 period

**DOI:** 10.1016/j.heliyon.2023.e12809

**Published:** 2023-01-05

**Authors:** Bao Cong Nguyen To, Bao Khac Quoc Nguyen, Tam Van Thien Nguyen

**Affiliations:** University of Economics Ho Chi Minh City (UEH), Ho Chi Minh City, Viet Nam

**Keywords:** COVID-19, Stock market volatility, Vaccine campaign, Vaccine initiation rate

## Abstract

During the COVID-19 pandemic, the news of clinical trials for vaccines and mass vaccinations have brought renewed optimism for stabilizing the economy and financial markets. However, the mental stress of investors or doubt about the effectiveness of government policies to cope with economic disruptions has caused stock market volatility. We investigate the significance of the vaccination rate in alleviating the global stock market volatility which is measured by the GJR–GARCH model. We discover that a higher vaccine initiation rate has a positive effect on global stock markets, especially in developed countries and areas with higher rates than their average. Our findings remain reliable even when using different projected volatility models and other estimates of the main independent variables. Mass immunization also implies that governments will not have to take extreme measures to handle the pandemic, which alleviates investor worries about compliance and the prolonged effects of COVID-19. Our research indicates that global stock markets are providing insight into the economic value of the development of COVID-19 vaccines, even before public vaccinations start.

## Highlights

• The COVID-19 mass vaccinations markedly mitigate volatility in the international stock markets.
• The impact of COVID-19 vaccinations is more substantial in developed markets and nations with an above-average vaccine initiation rate.
• The mass vaccinations indicate a lower probability of rigorous government reactions to the COVID-19 pandemic and mitigate investors' fears.
• The non-pharmaceutical government interventions signal terrible news as a reflection of higher volatility in stock markets.
• The COVID-19 cases and deaths increasing make investors' reactions to the trigger boosted uncertainty in the stock markets significantly.

## 1 Introduction

The COVID-19 pandemic appeared in late 2019 and started spreading in early 2020 in Wuhan, China creating an unprecedented shock to the global economy. COVID-19 has severely affected the economy of all countries, including developed countries, on both supply and demand sides, disrupting global supply chains and thereby slowing down the world economy [1,2]. When the world is struggling to cope with the pandemic, and the “zero COVID-19” approach seems ineffective, the announcements on clinical trials for COVID-19 vaccines and massive vaccinations as a way to live with COVID-19 bring back hope for stabilizing the economy, especially the financial markets.

As a result, COVID-19 has become a great interest in academic research. Much research focused on the pandemic’s early-stage consequences on economies and government responses [3,4,5]. Besides, since March 2020, when the epidemic spread globally, the stock markets of most countries have witnessed continuous significant declines and fluctuations [6]. Therefore, numerous studies searched for the reasons and have provided common evidence on the factors that affect stock market volatility, such as the infections cases, the number of deaths, government interventions (lockdown, stimulus packages), consumer behavior, and investors' fear [7–12].

The mental anxiety of citizens or uncertainty about the effectiveness of the government’s policies to deal with economic disturbances represents the main drive that leads to the fluctuations in the financial markets as investors' fear increases. Several studies indicate that increasing confirmed cases and deaths can generate more doubts about the efficiency of containment measures, which is conducive to higher volatility in the stock market [5,11,[13], [14], [15]]. Besides, the rise in recovery cases reduces uncertainty [16]. Moreover, other studies using other proxies such as the pandemic fear index, world uncertainty index, and economic policy uncertainty index to investigate this relationship have revealed the same conclusion [8,17,18].

To mitigate the adverse effects of COVID-19, governments have used fiscal and monetary policies. To be specific, many restrictive measures (school closures, lockdowns, etc.) have been implemented. In addition, central banks also cut interest rates near zero, reducing reserve ratios or expanding repurchase activities to limit the damage caused by this pandemic. Previous studies have shown that these measures contributed to somewhat softening and revitalizing the global stock markets [19,20]. These studies use a stringency index to examine the relationship between policy performance and the stock market or investigate fiscal spending effectiveness. Some studies proved that changes in government interventions could increase uncertainty, creating more volatility in the stock market [3,12,14,21,22].

Furthermore, new strains like Delta and MU have threatened the global effort. Government intervention becomes more costly and less effective in bringing the world economy back to its original state. Therefore, mass vaccinations play a cruel role in supporting these solutions. The new announcements about the emergence and the progress of vaccines from the end of 2020 have created expectations for recovery and the re-establishment of a new global normal. Many studies on the impact of vaccine development on the economy in general and the stock market have been carried out in a short time [16,23,24]. However, these studies mainly concentrate on vaccine announcements and the stock market’s stability. Therefore, our research^1^ is necessary since vaccine initiation rate variation among countries and the integration of global financial markets can question the accuracy of previous assessments. We contribute to the existing literature by testing how the difference in vaccine initiation rate impacts the global stock market’s reaction in general and the investors' behaviors in particular.

Our study examines the relationship between vaccine initiation rate and the stock market volatility in 32 emerging and developed countries from January 1, 2020, to June 24, 2022, via panel data regressions. Our main findings show that an increase in the daily vaccination rate signals good news to investors and mitigates the volatility of the global stock market. This implies that vaccines can help to reduce the symptoms, the government tighten measures, and start a period of safe living with COVID-19. These results are also consistent among developed and emerging markets. Meanwhile, a relative increase in non-pharmaceutical government involvement and the number of infections and deaths lead to higher volatility. These changes are considered bad news because they reflect the ability to control the pandemic and the economic health, causing investors' compliance fears about the likely enduring adverse effect of COVID-19. Moreover, our conclusions remain solid even when employing different projected conditional volatility models and alternate estimates of the critical, independent variables.

The paper is presented as follows: Section 2 consists of our methodologies; in Section 3, we detail the sample and descriptive statistics; In section 4, we present the findings and touch on the final thoughts in Section 5.

## 2 Methodologies

### 2.1 Modeling volatility

The GARCH model [25,26] was further advanced from the ARCH model of [27] in developing better-estimated models of conditional volatility. The AR, MA, and ARMA models are unconditionally heteroskedastic [28] since they are not responsible for volatility clustering and leptokurtosis, making them, in contrast to the typical GARCH models which reflect the asymmetric distribution of financial data.

According to Black’s [29] findings, there is a negative relationship between stock returns and changes in return instability. This implies that the volatility at time t is more affected by negative shocks (bad news) at time t-1 than positive shocks (good news). Due to the greater risk caused by the increased leverage brought on by a negative shock, this absence of symmetrical distribution is known as the leverage effect [25,30,31]. GARCH models appear to ignore the above occurrence because they assume that the magnitude of excess returns, rather than the positive or negative sign, affects conditional volatility. Furthermore, non-negativity restrictions may be broken since volatility is unstable, posing additional issues in estimating GARCH models.

Glosten et al. [32] and Glosten et al. [33] created the Glosten-Jagannathan-Runkle-GARCH (GJR-GARCH) and exponential GARCH (EGARCH) models. Based on the adjustments included in the GARCH-M model, which emphasizes anomalies in volatility’s response to both positive and negative shocks, Glosten et al. [32] modify the EGARCH model. The risk-return tradeoff is a well-known investment principle in finance.

The asymmetric GJR-GARCH (p,q)^2^ model is utilized in this work to calculate the conditional variance of the stock market index return during the tragedy, commonly known as stock market volatility. p = 1 and q = 1 are selected since there are typically the best matches for financial time series [32,34]. The GJR-GARCH (1,1) model is fully described below:

(1) $$r_{t}^{SM}=\mu +\varepsilon_{t}$$

(2) $$h_{t}^{SM}=\omega +\left(\alpha +\gamma d_{t-1}\right)\varepsilon_{t-1}^{2}+\beta_{j}h_{t-1}^{SM}$$

where $r_{t}^{SM}$ is the nation’s daily stock market index return computed as $\ln\left(P_{t}/P_{t-1}\right)$, $\varepsilon_{t}$ represents a zero-mean white noise without requiring serial independence, $h_{t}^{SM}$ denotes the conditional forecast variance at time *t*; the asymmetric parameter represented by the symbol γ, and $d_{t-1}$ indicates a dummy variable, which is detailed as follows:

$$d_{t-1}∶=\left\{ \begin{array}{l} 0\\ 1 \end{array}\right.\begin{array}{c} \;\\ \; \end{array}\begin{array}{l} if\;r_{t-1}\geq \mu \\ if\;r_{t-1}<\mu \end{array}$$

$$d_{t-1}∶=\left\{ \begin{array}{l} 0\\ 1 \end{array}\right.\begin{array}{c} \;\\ \; \end{array}\begin{array}{l} if\;\varepsilon_{t-1}\geq 0\;\left(good\;news\right)\\ if{\;\varepsilon }_{t-1}<0\;\left(bad\;news\right) \end{array}$$

### 2.2 Research model

We investigate the effects of vaccine initiation rate and COVID-19-associated variables on stock market volatility during the pandemic using panel data analysis and the following equation:

(3) $${SVOL}_{i,t}=\beta_{0}+\beta_{1}{VIR}_{i,t}+\beta_{2}{CGRT}_{i,t}+\beta_{3}{CASES}_{i,t}+\beta_{4}{DEATHS}_{i,t}+\varphi V_{i,t}+\delta_{t}+\varepsilon_{i,t}$$

In which *i* denotes country and *t* presents time. $\beta_{0}$ is a fixed term. The dependent variable ${SVOL}_{i,t}$ presents the daily stock return volatility computed by the conditional variance obtained from Eqs. (1) and (2). The key independent variables are proxied to examine the effects of people vaccinated, stringency measures, and the government’s response to stock market volatility. In our study, these variables include the vaccine initiation rate (${VIR}_{i,t}$) measured as the number of new COVID-19 vaccines divided by the entire population; the daily relative change^3^ of the Oxford Covid-19 Government Response Tracker index (${CGRT}_{i,t}$) [21,35]; and the relative daily change of COVID-19 total cases and fatalities per million people $\left({CASES}_{i,t}\;and\;{DEATHS}_{i,t}\right)$ [7,[9], [10], [11], [12]].

$V_{i,t}$ presents a collection of control variables at the national level proposed by prior research [11,16,36] and other macroeconomic indicators. In our model, we included four variables: the total market capitalization’s natural logarithm (${MCAP}_{i,t}$) and the daily percentage change in the exchange rate (${ER}_{i,t})$. We also incorporate the volatility index linked to the systematic component of international financial markets (${VIX}_{t}$), and the oil price (${OP}_{t}$). δ*t* represents a dummy variable for the error term’s time-invariant nation unobserved daily fixed effects ${.\;\varepsilon }_{i,t}$ is an error term. Table 1, shows a comprehensive list of variables, definitions, calculations, and sources.

**Table 1:** Variable definitions.

Variables	Definitions	Sources
*Dependent variable*		
SVOL	The daily stock return volatility is estimated by the conditional variance extracted from the asymmetric GJR-GARCH (1,1).	https://www.investing.com/indices
*Explanatory variables related to COVID-19*		
VIR	The vaccine initiation rate is measured by dividing the new COVID-19 vaccinations by the total population.	https://ourworldindata.org/coronavirus
CGRT	The relative change on a daily basis of the Oxford Covid-19 Government Response Tracker (OxCGRT).	https://github.com/OxCGRT/covid-policy-tracker
CASES	The relative change on a daily basis of COVID-19 aggregate cases per million people.	https://ourworldindata.org/coronavirus
DEATHS	The relative change on a daily basis of COVID-19 aggregate mortalities per million people.	https://ourworldindata.org/coronavirus
Variables related to country-level control and macroeconomic variables		
MCAP	The natural logarithm of the total market capitalization in USD.	https://www.investing.com/indices
ER	Changes in the currency rate that occur each day as a percentage.	https://www.investing.com/currencies/single-currency-crosses
VIX	The CBOE volatility index’s daily relative movement indicates the market’s expectation of impending volatility as expressed by the prices of stock index option.	https://fred.stlouisfed.org/series/VIXCLS
OP	The relative change in oil prices on a daily basis based on WTI crude oil in US dollars per barrel	https://www.investing.com/commodities/crude-oil

To assess the study’s objectives, we used panel data regressions, which included pooled ordinary least squares (OLS), fixed-effects estimation (FE), and random-effects estimation (RE). Post-estimate tests like the F-test, Breusch-Pagan Lagrange Multiplier (LM) [37], and Hausman [38] are used to evaluate the models' validity before choosing the best estimation technique.

To check the robustness of our findings, we also use the explanatory variables of COVID-19 namely the relative daily change of new COVID-19 vaccinations to millions of people (VIR2). Besides, we also use the relative daily change of the Government Response Stringency Index (GRSI), and the COVID-19 Pandemic Fear Index^4^ (PFI) [17,18]. We conduct estimations of the daily stock return volatility carried out based on ARMA (1,1)-GJR-GARCH (1,1), GARCH (1,1), EGARCH (1,1), TARCH/ZARCH (1,1).

## 3 Data and descriptive statistics

Our raw dataset includes the country’s daily stock market index,^5^ the explanatory variables as new COVID-19 vaccinations, Oxford Covid-19 Government Response Tracker (OxCGRT), the number of confirmed cases and fatalities, and the nation-level control and macroeconomic variables that span from January 1, 2020,^6^ to June 24, 2022. The study excludes unavailable observations and non-trading days. We built unbalanced panel data of 32 countries, comprising 13,709 country-day observations.^7^ To minimize the outliers' impact, some country-day observation variables were “winsorized” at the top and bottom 1% levels. An overview of the data about the descriptive statistics for the variables used in our study is presented in Table 2.

**Table 2:** Descriptive statistics.

Variables	Obs.	Mean	Min.	Max.	Std. Dev.	Skew.	Kurt.
SVOL	13,709	1.4136	0.5869	5.5380	0.5671	2.0719	10.209
VIR	13,709	0.0027	0.0000	0.0272	0.0039	1.7462	5.9489
CGRT	13,709	0.0002	−0.4823	0.5335	0.0246	−1.4865	114.19
CASES	13,709	0.0134	−0.0585	1.1124	0.0408	10.779	177.49
DEATHS	13,709	0.0140	−0.0136	3.0714	0.0740	20.390	648.38
MCAP	13,709	27.821	13.953	33.092	2.7998	−0.4766	2.8816
ER	13,709	−0.0001	−0.1123	0.0862	0.0061	−0.5770	23.765
VIX	13,709	0.0168	−1.0178	1.8152	0.2281	4.2265	27.829
OP	13,709	0.0010	−0.3024	0.1660	0.0413	−1.8649	17.203

Table 2 reports the summary descriptive statistics for the main variables used in this research. Over the period studied, the SVOL variable’s mean for 32 nations is 1.4136 and fluctuated widely, with minimum and maximum values reaching 0.5869 and 5.5380, respectively. The standard deviation of this measure is 0.5671, indicating significant volatility. A possible effect of the vaccine initiation rate (VIR) is reported at 0.0027 in the mean value, and the VIR has changed from zero to 0.0272. In the early stage, this variable is almost equal to 0 since the vaccination supply is still limited in some countries. The range of values for the government’s reactions to COVID-19 assessed by the CGRT variable is −0.4823 to 0.5335. Furthermore, the mean daily relative change in total confirmed cases (CASES) and fatalities (DEATHS) per million people is 0.0134 and 0.0140.

## 4 Results

Regression results of Eq. (3) in Table 3 provide evidence that the vaccine initiation rate (VIR) reduces volatility in stock markets (SVOL) of 32 countries during COVID-19. Statistical evidence from our research sample revealed a negative connection between VIR and SVOL at the statistical significance level of 1%. This relation is also genuine when considering Eq. (3) with the emerging markets (EMs) in column (ii) and developed markets (DMs) in column (iii), or the sample related to the changes in VIR is above its overall average (VIR_Above) and otherwise. Moreover, the relationship between VIR and SVOL is stronger within DMs and countries having VIR_Above than in the rest of the total sample. The substantial implications of these results show that boosting new COVID-19 vaccinations positively impacts mitigating volatility in the global stock markets. The findings are consistent with prior studies on the co-movements of the stock markets and tragedy fears. This relationship turns weaker and has more minor fluctuations in the correlations with the announcement of the mRNA-based COVID-19 vaccines [18], implying that the COVID-19 vaccinations assist in stabilizing the global equity market [23,[39], [40], [41]]. However, our results are the opposite of the study conducted by Bakry et al. [16] regarding Pfizer-BioNTech’s successfully developing a COVID-19 vaccine which makes volatility in stock markets increase, although this implies a significant positive development.

**Table 3:** Impact of vaccine initiation rate and variables related to the pandemic on stock market volatility during COVID-19.

Dependent Variable = SVOL	All	EMs	DMs	VIR_Above	VIR_Below
	(i) RE	(ii) RE	(iii) RE	(iv) RE	(v) RE
VIR	−0.1293*** (−5.18)	−0.1036*** (−2.88)	−0.1325*** (−4.90)	−0.1394*** (−5.26)	−0.0851 (−1.21)
CGRT	0.5213** (2.07)	0.6497 (1.48)	0.3849 (1.30)	0.8356** (2.45)	−0.1164 (−0.44)
CASES	2.9244*** (4.67)	3.0053** (2.35)	2.7242*** (3.98)	3.4129*** (4.01)	2.1121** (2.54)
DEATHS	1.4065*** (4.88)	1.1424*** (3.07)	1.6713*** (3.70)	1.4021*** (3.92)	1.4144*** (2.94)
MCAP	0.0602** (2.00)	0.0112 (0.35)	0.0933* (1.80)	0.0523* (1.69)	0.0785 (1.11)
ER	−1.2386 (−1.17)	−0.6315 (−0.41)	−2.1093* (−1.81)	−2.0252** (−2.13)	−0.2437 (−0.12)
VIX	−0.1254*** (−5.30)	−0.0761*** (−2.77)	−0.1648*** (−5.06)	−0.1443*** (−4.81)	−0.0865 (−2.42)
OP	−0.2261*** (−4.01)	−0.1856*** (−2.81)	−0.2549*** (−2.99)	−0.2896*** (−3.98)	−0.0742 (−1.23)
Constant	−0.2949 (−0.36)	1.0618 (1.23)	−1.1934 (−0.85)	−0.1550 (−0.18)	−0.6167 (−0.33)
Day dummies	Yes	Yes	Yes	Yes	Yes
N. observ.	13,709	5921	7788	9332	4377
N. countries	32	14	18	22	10
R2	0.2259	0.1767	0.2674	0.2544	0.1732
F	–	–	–	–	–
λ2	101.39***	64.46***	127.16***	94.23***	92.31***
Hausman test	5.20	11.60	8.61	3.97	0.65
LM test	4.8e+05***	3.5e+05***	1.2e+05***	2.0e+05***	2.2e+05***

*Note:* This table summarizes the findings of Eq. (3). Column (i) presents our empirical investigation of the COVID-19 pandemic in 32 countries. The rest of our research is based on a subset of the emerging markets (EMs) and developed markets (DMs) given in columns (ii) and (iii). Columns (iv) and (v) report the results of the sample-related VIR_Above when the changes in the vaccine initiation rate (VIR) go beyond its overall average. Simultaneously, VIR_Below connotes when the VIR changes below its average value. In addition, to increase the readability of our tables, VIR is multiplied by 100. The values of the T statistic are given in parentheses. The symbols ***, **, and * represent the statistical significance of the coefficients at 1%, 5%, and 10%, respectively. Day dummies are included in all specifications. N.observ., N.countries and R^2^ present, respectively, the observations, nations, and an adjusted coefficient of determination.

We also examine the impact of the change in the government’s response (CGRT), COVID-19 total cases, and fatalities (CASES and DEATHS) on stock market volatility. Statistical evidence in Table 3 illustrates a significant contrast in the positive relationship between these variables and SVOL. The above results show that non-pharmaceutical government involvement increases stock market volatility. This indicates that the government’s actions signal bad news as a likeness of the potential of pandemic control. Low levels of trust in governments' responses cause compliance doubt and fear for investors that the adverse consequences of COVID-19 would be more prolonged, particularly for emerging markets. A significant and correlated association exists between shifts in the total COVID-19 cases and deaths and the fluctuations in the stock market. Confirmed cases related details are more important than deaths when looking at the results from Table 3. A consistent view from these results shows that the investors' reactions to the trigger elevated uncertainty about how critical the pandemic may get. These findings are similar to recent studies [11,12,[14], [15], [16],^42^]. Lastly, our model’s control variables are all statistically significant, which aligns with prior research. Table 4 demonstrates that our outcomes remain robust when different calculations of main independent variables and predicted conditional volatility models are used.

**Table 4:** Robustness tests.

No.	Robustness check	VIR	CGRT	CASES	DEATHS
(1)	_VIR is proxied by the daily relative change of the new COVID-19 vaccinations divided by million people (VIR2).	−0.0585* (−1.69)			
	_CGRT is proxied by the Government Response Stringency Index (GRSI).		0.3826*** (4.30)		
	_CASES and DEATHS are proxied by the daily relative change of the COVID-19 Pandemic Fear Index (PFI).			−0.0117** (−2.28)	
(2)	SVOL based on ARMA (1,1)-GJR-GARCH (1,1) model	−0.1329*** (−5.63)	0.4705* (1.90)	2.8825*** (4.74)	1.3490*** (4.78)
(3)	SVOL based on GARCH (1,1) model	−0.1184*** (−3.81)	0.3984 (1.01)	2.8692*** (4.51)	1.2074*** (4.36)
(4)	SVOL based on EGARCH (1,1) model	−0.1158*** (−2.79)	0.9053 (1.44)	3.5588*** (2.74)	0.8583*** (2.59)
(5)	SVOL based on TARCH/ZARCH (1,1) model	−0.1660*** (−6.43)	0.3334 (1.54)	2.9117*** (5.04)	1.1120*** (4.55)

*Note:* This table summarizes the findings of Eq. (3), which used panel data regressions to check the robustness of our findings to alternative measures of the main variables and estimated models of conditional volatility. In addition, to increase the readability of our tables, VIR is multiplied by 100. The values of the T statistic are given in parentheses. The symbols ***, **, and * represent the statistical significance of the coefficients at 1%, 5%, and 10%, respectively. The results of the control variables and model tests are not disclosed but are accessible from the authors upon request.

## 5 Conclusion and policy recommendation

Our results indicate that in the context of COVID-19, mass vaccination significantly decreases the stock market volatility in both groups of countries. This result implies that an increase in the population immunized contributes to stabilizing the stock market by creating optimism and boosting investor confidence. We also found that this effect is more potent in developed and emerging countries. Our robustness test applying another proxy for the vaccine initiation rate and employing different conditional volatility models came to the same conclusion. In addition, we also found that the government’s non-pharmaceutical interventions can generate higher volatility in the stock market since these measures might reflect a prolonged and negative impact of COVID-19 and hence induce a low level of trust in the government actions along with a rise of uncertainty from citizens in general and investors. Finally, we also indicate that the movement of infections and death cases creates more-than-proportional stock market volatility.

Our research has resulted in a number of practical implications that should be taken into account by various stakeholders. Investors need to keep an eye on the vaccination policies and progress of countries worldwide, as they vary in terms of vaccination levels. Additionally, investors should track the efficacy of current vaccinations against new COVID-19 variants, as a decrease in efficacy could lead to a decline in vaccination rates and the need for a booster program. This could mean that investors need to adjust their portfolios based on these developments, taking into account the different effects of vaccinations in both developed and emerging markets. Policymakers should also recognize the importance of vaccinations, and create long-term solutions to ensure sustainable development after the pandemic, as well as stabilize the stock and financial markets.

The main limitations of our research are that it does not take into account other economic issues such as inflation, interest rates, GDP change, and so on. It is undeniable that vaccination development and mass vaccination represent a topic that needs further attention from researchers using different approaches to figure out the role of COVID-19 vaccines in the international financial markets. Although the inclusion of macroeconomic variables is significant, we leave it for future research.

## Author contribution statement

Bao Cong Nguyen To; Bao Khac Quoc Nguyen; Tam Van Thien Nguyen: Conceived and designed the experiments; Performed the experiments; Analyzed and interpreted the data; Contributed reagents, materials, analysis tools or data; Wrote the paper.

## Funding statement

Mr. Bao Cong Nguyen To, Bao Khac Quoc Nguyen, Tam Van Thien Nguyen was supported by University of Economics Ho Chi Minh City (UEH) (2021-10-01-0607).

## Data availability statement

Data will be made available on request.

## Declaration of interest’s statement

The authors declare no competing interests.

## Additional information

No additional information is available for this paper.

## Figures and Tables

**Table:** 

Panel A: Emerging Markets (EMs)			Panel B: Developed Markets (DMs)		
No.	Country	Stock Index	No.	Country	Stock Index
1.	Brazil (BRA)	BOVESPA	1.	Australia (AUS)	S&P_ASX 200
2.	China (CHN)	Shanghai Composite	2.	Austria (AUT)	ATX
3.	Greece (GRC)	Athex Composite	3.	Belgium (BEL)	BEL 20
4.	India (IND)	NIFTY 500	4.	Canada (CAN)	S&P_TSX
5.	Indonesia (IDN)	IDX Composite	5.	France (FRA)	CAC 40
6.	Malaysia (MYS)	FTSE KLCI	6.	Germany (DEU)	DAX30
7.	Mexico (MEX)	S&P_BMV IPC	7.	Hong Kong (HKG)	Hang Seng
8.	Philippines (PHL)	PSEi Composite	8.	Ireland (IRL)	ISEQ Overall
9.	Poland (POL)	WIG 30	9.	Israel (ISR)	TA125
10.	Qatar (QAT)	QE General	10.	Italy (ITA)	FTSE Italia All Share
11.	South Africa (ZAF)	FTSE_JSE All Share	11.	Japan (JPN)	Nikkei 225
12.	South Korea (KOR)	KOSPI Composite	12.	Netherlands (NLD)	AEX
13.	Thailand (THA)	SETI	13.	Norway (NOR)	OBX
14.	Turkey (TUR)	BIST 100	14.	Portugal (PRT)	PSI 20
			15.	Spain (ESP)	IBEX 35
			16.	Switzerland (CHE)	SMI
			17.	United Kingdom (GBR)	FTSE 100
			18.	United States (USA)	S&P 500

## Footnotes

1 Our research was conducted independently in April 2021, based on our understanding of the research gap existing at the time. We had anticipated that our results would be the first evidence of the relationship between COVID-19 vaccines and stock market volatility until Rouatbi et al.’s [21] paper was released on June 24, 2021. Although the two studies have different methodologies, this diversity is an asset to research as it adds to the discussion surrounding how stock markets react to bad news and good news during the pandemic.

2 Furthermore, Akaike Information Criterion (AIC) or Bayesian Information Criterion (BIC) can be adopted to select the optimal model (order p and q) (AIC). p presents the order of the GARCH terms and q presents the order of the ARCH terms.

3 The midpoint relative change ($\Delta x/\bar{x}$*)* was used to avoid issues related to infinite percentage changes when events such as COVID-19 first appeared.

4 ${PFI}_{i,t}=0.5\times \left[\frac{{Cases}_{i,t}}{{Cases}_{i,t}+{Cases}_{i,t-14}}+\frac{{Deaths}_{i,t}}{{Deaths}_{i,t}+{Deaths}_{i,t-14}}\right]\times 100$

5 Appendix A reports details on the stock market index chosen for each country in our sample.

6 The time during which the pandemic was first spreading.

7 Before running empirical models, the dataset must guarantee that the unit root problem is avoided, and we employ the first or second difference of the data as needed. The Fisher-type tests of Augmented Dickey-Fuller (ADF) and Phillips-Perron (PP) are sufficient to establish that our variables are stationary for the unbalanced panel data set (Choi, 2001) 43. The findings of these tests have not been published but are accessible from the authors upon request.
